# Supplementary material for: Complexome profiling of the Chlamydomonas psb28 mutant reveals TEF5 as an early PSII assembly factor
Source: Plant Cell. 2025 Mar 17;37(6):koaf055. doi: 10.1093/plcell/koaf055 (PMC12164590; doi:10.1093/plcell/koaf055)
Supplement: koaf055_Supplementary_Data [file koaf055_supplementary_data.zip › psb28_interactive.zip!/plots/intensity/qualityPlots/peptides/CGL63_Cre10.g444800.t1.2.html]

### Cre10.g444800.t1.2

Trivial name: CGL63  
SeqCoverage [median]: 10.00  
Mass: 27.89 kDa  
  
 

| Sequence | MissedCleavage | Mass [Da] | wtIntensity | muIntensity | Score |
| --- | --- | --- | --- | --- | --- |
| AAAGVLEDLR | 0 | 1013.55 | 1022.55 | 0.00 | 67.99 |
| TALQQYIYR | 0 | 1154.61 | 0.00 | 854.35 | 66.68 |
|  |  |  | 1022.6 | 854.4 |  |
